# Insights into the salt levels in bread offers in Slovenia: trends and differences

**DOI:** 10.3389/fnut.2024.1473362

**Published:** 2025-01-14

**Authors:** Saša Kugler, Hristo Hristov, Urška Blaznik, Maša Hribar, Edvina Hafner, Anita Kušar, Igor Pravst

**Affiliations:** ^1^National Institute of Public Health, Ljubljana, Slovenia; ^2^Faculty of Medicine, University of Ljubljana, Ljubljana, Slovenia; ^3^Nutrition Institute, Ljubljana, Slovenia; ^4^Biotechnical Faculty, University of Ljubljana, Ljubljana, Slovenia; ^5^VIST–Faculty of Applied Sciences, Ljubljana, Slovenia

**Keywords:** bread, sodium, salt, ICP-MS, Slovenia

## Abstract

**Objective:**

Bakery products are considered as one of main dietary sources of sodium/salt in Slovenia. Our main objective was to assess the salt content in bread in Slovenia, focusing into different bread categories and sales channels. The data collected in 2022 was compared with year 2012.

**Methods:**

A follow-up study on salt content of bread sold in Slovenia was conducted. Bread samples were purchased in large retail shops and smaller bakeries across 11 statistical regions of Slovenia. Sodium content was determined by inductively coupled plasma mass spectrometry; salt content was calculated by multiplying sodium content with 2.54, assuming all sodium corresponds to sodium chloride.

**Results:**

In 2022, 178 bread samples were purchased and analyzed. Weighted mean salt content in bread was 1.35 (95% CI 1.28–1.42) g/100 g in 2012, and 1.26 (95% CI 1.22–1.29) g/100 g in 2022, showing a 7% decrease. Notable differences in the salt content were observed between various bread subcategories and retail environments. In addition, a significant difference was observed between white wheat bread sold in large retail shops and smaller bakeries, where a higher salt content was observed.

**Conclusion:**

While study results show small decrease in the salt content in bread in Slovenia in last decade, the salt reduction targets set by the WHO have not been met. Additional efforts are needed to stimulate bread reformulation with reducing salt content.

## 1 Introduction

Sodium is one of the major cations in extracellular fluid. It enables proper signal transduction, muscle contraction and helps to regulate fluid balance in the body (1). While its role in the body is important, current intakes of sodium are much higher than needed or recommended, with the current global estimate of 3.95 g sodium/day (equivalent to 10.06 g salt/day), which is nearly twice the World Health Organization (WHO) recommended limit of 2 g sodium/day (equivalent to 5 g salt/day) (2). High sodium (salt) intake has been associated with the risk for developing gastric cancer (3, 4), and higher urinary calcium excretion, thereby increasing risk of osteoporosis and kidney stones (4). However, the most notable association, is with high blood pressure. Several systematic reviews and meta-analyses have shown that reducing sodium intake can lead to a reduction in systolic blood pressure (5, 6) and risk of cardiovascular disease (7). It has also been recognized as the most cost-effective strategy in reducing cardiovascular events and mortality rates (8, 9).

In 2013, the WHO published the Global action plan for prevention and control of noncommunicable diseases 2013–2020, which included a goal of 30% relative reduction in sodium/salt intake (10). To achieve this, comprehensive programs which engage different sectors (e.g., food manufacturers), as well as consumers, are needed. According to a systematic review by Trieu et al., majority of countries with salt reduction strategies use multifaceted approaches, implementing mainly food reformulation, consumer education, front-of-package labeling, interventions in public institution settings, and taxation (11). Most of these strategies have been recognized by the WHO as best-buy interventions for reducing sodium intake (12).

Reducing consumption of highly processed foods and food reformulation, are suggested as crucial for reducing sodium intake in the population (13, 14). This is understandable since processed foods represent about 75% of sodium intake in European and North American countries (1, 15). While specific food categories contributing most to sodium intake slightly vary between countries, cereals and cereal-based foods, and meat products are often among top contributors. This has been reported in countries such as United Kingdom and United States (16), and similarly in France (17) and New Zealand (18). Among cereal-based foods, bread and bakery products are suggested as main sources of salt in European countries (19). According to a recent systematic review of dietary sodium sources worldwide, they account for 25 to 40% of sodium contribution in many European countries and the United States (20). Bread and bakery products are therefore among main targets for food reformulation.

Sodium has an important role in bread, impacting both sensory characteristics and technological aspects, such as development of gluten structure, fermentation, mixing and baking (21, 22). Despite this, a gradual reduction of sodium content can be achieved (19, 23). An example of successful reduction was the United Kingdom, where sodium content in pre-packed bread was reduced by about 20% between 2001 and 2011 (24).

Since bread and bakery products were also recognized as the main contributors to sodium intake in Slovenia (25), efforts were made to reduce its content by setting voluntary sodium reduction targets (26). A recent study has shown limited improvement in sodium content in pre-packed foods, including pre-packed bread, between 2011 and 2020 (27). Similar results were also suggested for non-prepacked bread sold in retail shops in 2012 (28), however there has been no subsequent monitoring of these bread types since then.

Therefore, the main objective of this study was to assess the change in sodium/salt content of bread between 2012 and 2022. For year 2022 we additionally compare sodium/salt content in white wheat bread sold in small bakeries and large retail stores, and in different types (prepacked and unpacked), and different bread categories (white wheat, mixed and wheat toast). With consideration that the European Union regulations mandate food labeling of sodium in form of salt content, herein reported results are also presented for salt content, although all samples were analyzed for sodium content. Salt content was calculated assuming all sodium corresponds to sodium chloride.

## 2 Materials and methods

### 2.1 Sample collection

We conducted a longitudinal follow-up study investigating the salt content in bread available in Slovenian food supply, replicating a previous study published in 2012 (28). Bread samples were collected between November and December 2022 from large retail shops and smaller bakeries across Slovenia. In contrast to the 2012 study, which focused on bakeries and retail shops in the capital city metropolitan area, the 2022 sampling strategy incorporated suppliers in different statistical regions of Slovenia.

While an ideal bread sampling strategy would involve determining the number of breads based on accurate market share data for individual products within specific bread categories, such data was not readily available. Therefore, we utilized aggregate bread consumption estimates from household surveys of the Statistical Office of the Republic of Slovenia (SURS) conducted in 2010 and 2018, along with market share estimates for individual manufacturers, to determine the appropriate number of bread samples for each bread category. Similar to the sampling process employed in 2012, which prioritized bread types with higher consumption shares, as reported in the national survey results for household bread consumption in 2010, the 2022 sampling considered data from the bread type consumption survey as provided in the 2018 households’ consumption report (29). Power analysis was performed with aim to determine the required sample size for detecting a statistically significant 10% reduction in salt content in retail white wheat bread between 2012 and 2022. This analysis utilized an effect size of 0.85, a significance level of 0.05, and a desired power of 0.8, with an allocation ratio of 2:1 (in comparison to 2012 sampling). The estimated sample size for white wheat bread in 2022 was N = 28. Sample size for other bread categories was recalculated with weighting-approach using the last available households’ consumption report (29).

Regarding sampling in large retail shops, the sampling process stipulated that bread samples must originate from manufacturers and retailers selling bread across the country, to ensure comprehensive representation of the bread market. Retail shops affiliated with retail chains exceeding a 5% market share in the country’s total food and other staple items sales, and bread manufacturers selling within these retail shops and maintaining at least 30 permanent employees and €2 million in annual revenues, were included in the sampling process. Additionally, breads with the highest turnover within the selected bread categories, as identified by shop assistants stationed at the bread-selling desks, were included in the sample. The 2022 sampling also encompassed breads listed on the government’s price monitoring platform (30), which tracks bread prices to address concerns about rising food costs.

While the sampling conducted in the smaller bakeries in 2012 included only stores located in the metropolitan area (28), the sampling in 2022 was conducted in bakeries located in 11 official statistical regions of Slovenia, according to the Nomenclature of Territorial Units for Statistics (NUTS) - level 3 classification (31). This accounted for regions with approximately 97% of the country’s population. Only one of the smallest regions (Lower Sava) was not included, due to logistic constraints. Bakery inclusion was based on their affiliation to the specific region and information obtained from Google Maps, which provided the names and locations of eligible bakeries. For each region, a separate sampling pool was established, and two bakeries were randomly selected. We purchased one white and one wholegrain bread sample with the highest turnover, as recommended by the shop assistant working in the bakery store. Purchase of samples was done in neutral way, with researcher acting as an ordinary customer, asking for “bestselling white bread” and “bestselling wholegrain bread”.

The combined estimated market share of all retail shops and bakery stores included in the bread selection for both years is estimated at >85%. Additionally, the bread manufacturers included in the study were estimated to cover at least 75% of all bread produced and sold in the country. The estimated market shares were obtained through publicly available sales data from retail shops and bread manufacturers for the sampling years.

While in 2012 altogether 45 bread samples were purchased (41 in large retail shops and 4 in small bakeries), the 2022 sampling included not only bread, but also toast bread and small bread rolls. The total sample in 2022 included 178 bread products, with 117 sampled in large retail shops and 61 in smaller bakeries. The decision for such increase in the sampling was done with consideration of the variability in the salt content observed in certain bread categories in the 2012 study. The data presenting the distribution of samples in selected bread categories per sampling year are provided in the Supplementary Table S1. The 2012 sampling included 14 white wheat bread samples, 9 half-white wheat breads, 6 dark breads, 16 mixed and 3 rye breads, while 2022 sample consist of 60 samples of white wheat breads, from which 28 were provided by small bakeries and 32 from large retail shops, 15 half-white wheat breads, 16 dark wheat breads, 29 mixed breads, 3 rye breads, 33 wholegrain breads (29 from smaller bakeries and 4 from large retail shops), 15 small white wheat bread rolls and 10 white wheat toast breads. Samples were categorized according to Slovenian Rules on quality of bakery products (32), which defines mixed wheat bread as bread made from different types of flour, with at least 51% of wheat flour. Rye bread is defined as bread made from rye flour, with up to 20% wheat flour and wholegrain wheat bread as bread containing at least 80% wholegrain wheat flour. The difference between white, half-white and dark wheat bread is mainly in the mineral (ash) content of the used wheat flour (33).

### 2.2 Sample preparation and sodium content determination

After purchase the bread samples were packaged in plastic bags, stored at room temperature (~23°C), and delivered to the laboratory.

The sodium content of bread samples in 2022 was determined in accredited laboratory Mérieux NutriSciences (Resana, Italy) using AOAC Official Method 2011.14 (34), utilizing quantification with inductively coupled plasma mass spectrometry (ICP-MS); results were expressed as mg/100 g. The sodium content in bread samples from 2012 was determined by potentiometric method, which quantifies sodium chloride content via chloride ions; the method uses silver nitrate solution as titrant (28). Salt content of bread samples was calculated by multiplying sodium content with 2.54, assuming all sodium corresponds to sodium chloride (NaCl).

### 2.3 Statistical analysis

Based on product type, each sample was assigned to a category as defined by national Rules on quality of bakery products (32). To draw conclusions on the mean salt content in Slovenia for overall category of bread, we used a weighting approach with consideration of national SURS data from 2010 and 2018 on household bread consumption. Specific weights for all sampling units (e.g., bread categories) were assigned to avoid bias due to the sampling technique. The weights used are presented in Supplementary Table S1.

For all bread products per sampling year and each bread subcategory, the mean, median, standard deviation, range, standard error, and confidence intervals were used to describe the salt content in grams per 100 grams of product. A one-way ANOVA analysis was conducted to determine the mean difference in salt content between white wheat breads purchased from small bakeries and large retail shops. Paired comparison t-tests were used to identify differences in salt content between analytical measurements and reported values in nutrition declarations (labels or online – where applicable) for pre-packed and un-packed bread products, and across different bread types. Additionally, linear mixed model analysis was used to determine the difference in the salt content of bread using the same bread categories in both sampling years and per different type of stores. Values were considered as statistically significant when p was <0.05. For statistical analyses and data representation IBM SPSS Version 27 (IBM SPSS, IBM Corp., Armonk, NY) and GraphPad Prism (GraphPad Software, version 8.2.0., San Diego, United States) was used.

## 3 Results

Results of monitoring bread salt content in Slovenia in 2022, and comparison with 2012 data (25, 28) are presented in Table 1.

**Table 1:** Salt content of bread purchased in large retail shops and small bakeries across Slovenia in 2012 and 2022.

Type of bread	Purchase location	2012*				2022			
		*N* (%)	Mean (SD) salt content (g/100 g)	Median	Range	*N* (%)	Mean (SD) salt content (g/100 g)	Median	Range
White wheat bread	All	14 (31.1)	1.35 (0.25)	1.35	0.9	60 (33.7)	1.28 (0.20)	1.28	1.21
	Small bakeries	1	1.00			32	1.34 (0.21)	1.33	1.21
	Large retail shops	13	1.38 (0.24)	1.40	0.9	28	1.21 (0.16)	1.24	0.71
Half-white wheat bread	Large retail shops	9 (20.0)	1.37 (0.17)	1.40	0.6	12 (6.7)	1.24 (0.13)	1.21	0.51
Dark wheat bread	Large retail shops	6 (13.3)	1.32 (0.08)	1.30	0.2	16 (9.0)	1.15 (0.14)	1.12	0.53
Rye breads	Large retail shops	3 (6.7)	1.37 (0.06)	1.40	0.1	3 (1.7)	1.18 (0.13)	1.11	0.24
Mixed breads	All	13 (28.9)	1.36 (0.18)	1.40	0.7	29 (16.3)	1.22 (0.25)	1.15	0.97
	Small bakeries	3	1.50 (0.20)	1.50	0.4				
	Large retail shops	10	1.32 (0.16)	1.35	0.5				
Wholegrain**	All					33 (18.5)	1.36 (0.29)	1.33	1.44
	Small bakeries**					29	1.37 (0.30)	1.33	1.44
	Large retail shops					4	1.28 (0.24)	1.25	0.55
White wheat rolls	Large retail shops					15 (8.4)	1.29 (0.19)	1.34	0.66
White wheat toast bread	Large retail shops					10 (5.6)	1.36 (0.20)	1.39	0.55

SD, standard deviation. *Previous monitoring data was reported by Blaznik et al. (28) and Ribič et al. (25). **Wholegrain bread samples in small bakeries correspond to samples that were offered on the request for “bestselling wholegrain bread,” however, considering that samples were purchased as non-prepacked and without written declaration, these could be also mixed breads, not meeting strict regulatory requirements for wholegrain bread.

In 2012 the lowest mean salt content was measured for dark wheat bread (1.32 g/100 g). While the salt content of white wheat bread (1.35 g/100 g) was lower compared to rye or half-white wheat bread, the standard deviation and range were higher than in other types of bread.

In 2022, the lowest mean salt content was also measured in dark wheat bread (1.15 g/100 g), while the highest content was observed in white toast bread (1.36 g/100 g) and wholegrain bread from smaller bakeries (1.37 g/100 g). Results also show high variability and range in certain categories such as white wheat bread (in smaller bakeries), wholegrain wheat bread and mixed breads.

The unweighted and weighted data on salt content in breads sold in Slovenia for years 2012 and 2022 are presented in Supplementary Table S2. The unweighted data show that our sampling sufficiently covered different bread categories, considering statistical data on bread type consumption (SURS, 2018). Weighting was used to provide insights about the salt content in “typical” bread consumed in Slovenia using SURS statistical data about the consumption of various types of bread. Such imaginary typical bread is constructed with fixed proportions of various bread types, according to their consumption share. Study results show a decrease of 7% in the salt content of such typical bread from year 2012 to 2022. In addition, a decrease of 12% in the salt content was observed specifically in white wheat bread from large retail shops in the same period.

Table 2 shows results of linear mixed effects model comparison analysis using the same categories of breads sampled in 2012 and 2022 in small bakeries and large retail shops. The results show significant decrease in the mean salt content between breads sampled in year 2022 compared to 2012 in large retail shops. Additionally, although the similar trend was present in 2012, we evidenced significant difference in mean salt content in 2022 between the breads sold in small bakeries and those sold by large retail shops, where lower salt content was observed.

**Table 2:** Results of linear mixed effect models comparing salt content of bread purchased in different type of stores in Slovenia in 2012 and 2022.

Type of store	Year of sampling	Mean	SE (Mean)	Difference	95% CI (Diff.)		*z*	*p*
Small bakeries	2012	1.375	0.09	−0.037	−0.23	0.15	−0.38	n.s
	2022	1.338	0.03					
Large retail shops	2012	1.351	0.03	−0.158	−0.23	−0.09	−4.51	<0.001
	2022	1.193	0.02					
Small bakeries	2012	1.375	0.09	−0.024	−0.21	0.16	−0.25	n.s
Large retail shops		1.338	0.03					
Small bakeries	2022	1.351	0.03	−0.144	−0.22	−0.07	−3.80	<0.001
Large retail shops		1.193	0.02					

CI, confidence interval; n.s, not significant; SE, standard error.

**Figure 1:**
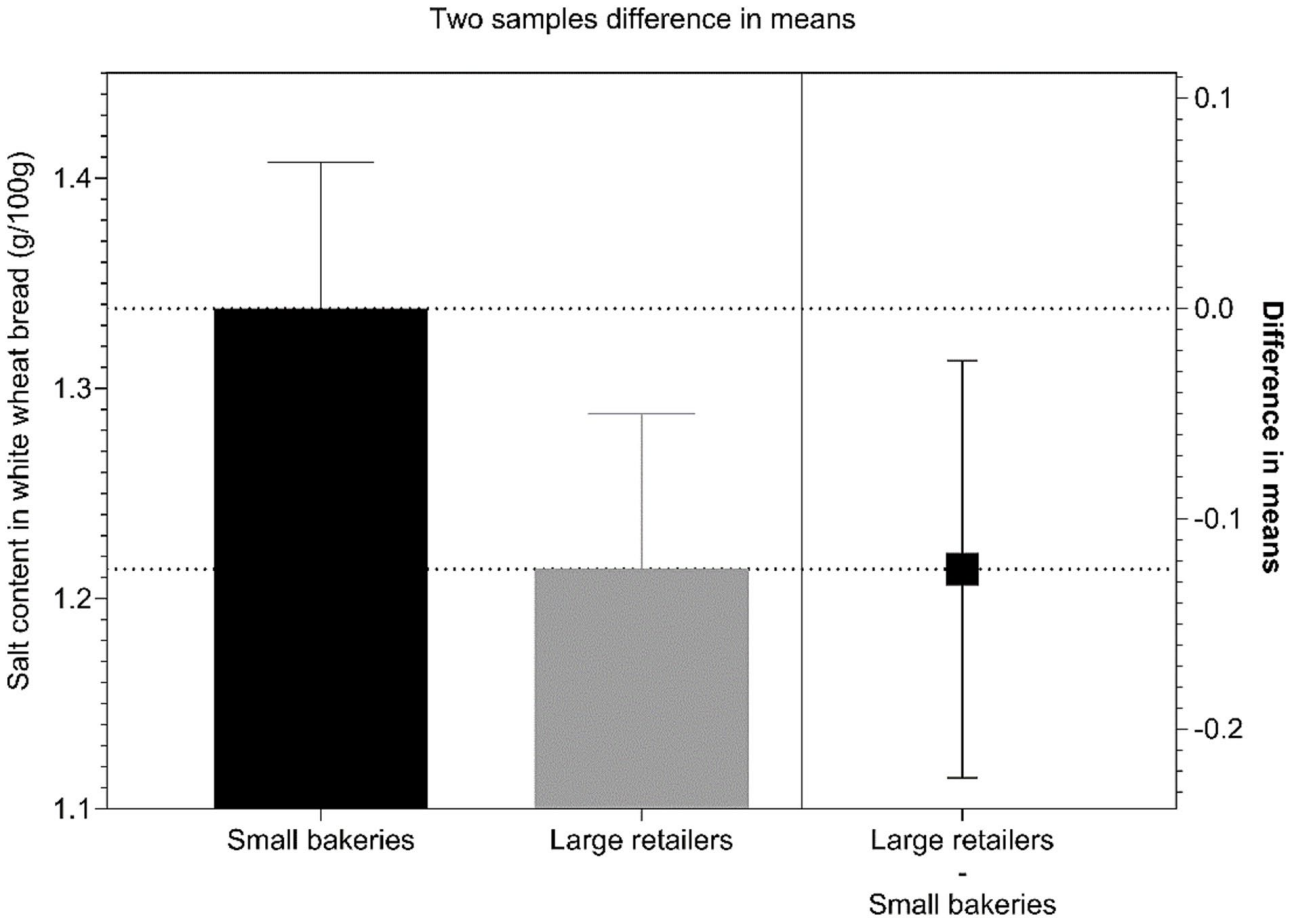
Analysis of differences in mean salt content (g/100 g) in white wheat breads purchased in small bakeries and large retail shops in 2022.

Using the one-way ANOVA and testing for between-subject effect related to type of retail environment, we found a significant difference (*p* = 0.015) between the salt content present in white wheat bread sold in small bakeries compared to white wheat bread sold in large retail stores, which commonly sell bread produced by large manufacturers. The white wheat bread sold in small bakeries has a mean content of 1.34 (0.21) g salt/100 g, while the one sold in large retail shops has mean content of 1.21 (0.16) g salt/100 g. The salt content found in wholegrain breads purchased in small bakeries (*N* = 29) has the mean content of 1.37 (0.30) g salt/100 g, while wholegrain breads sold in large retail shops (*N* = 4) have 1.28 (0.24) g salt/100 g. Although the difference in means for wholegrain breads implies significant difference, the small sample size of samples purchased in large retail stores and the large variability within the sample obtained in small bakeries, did not provide the resource to further explore the observable difference. It should be mentioned that wholegrain bread samples in small bakeries correspond to samples that were offered by bakery staff when we asked for “bestselling wholegrain bread.” These samples were non-prepacked and without any written declaration on the content of wholegrain ingredients and might not actually meet strict regulatory requirements for wholegrain bread (Figure 1).

**Figure 2:**
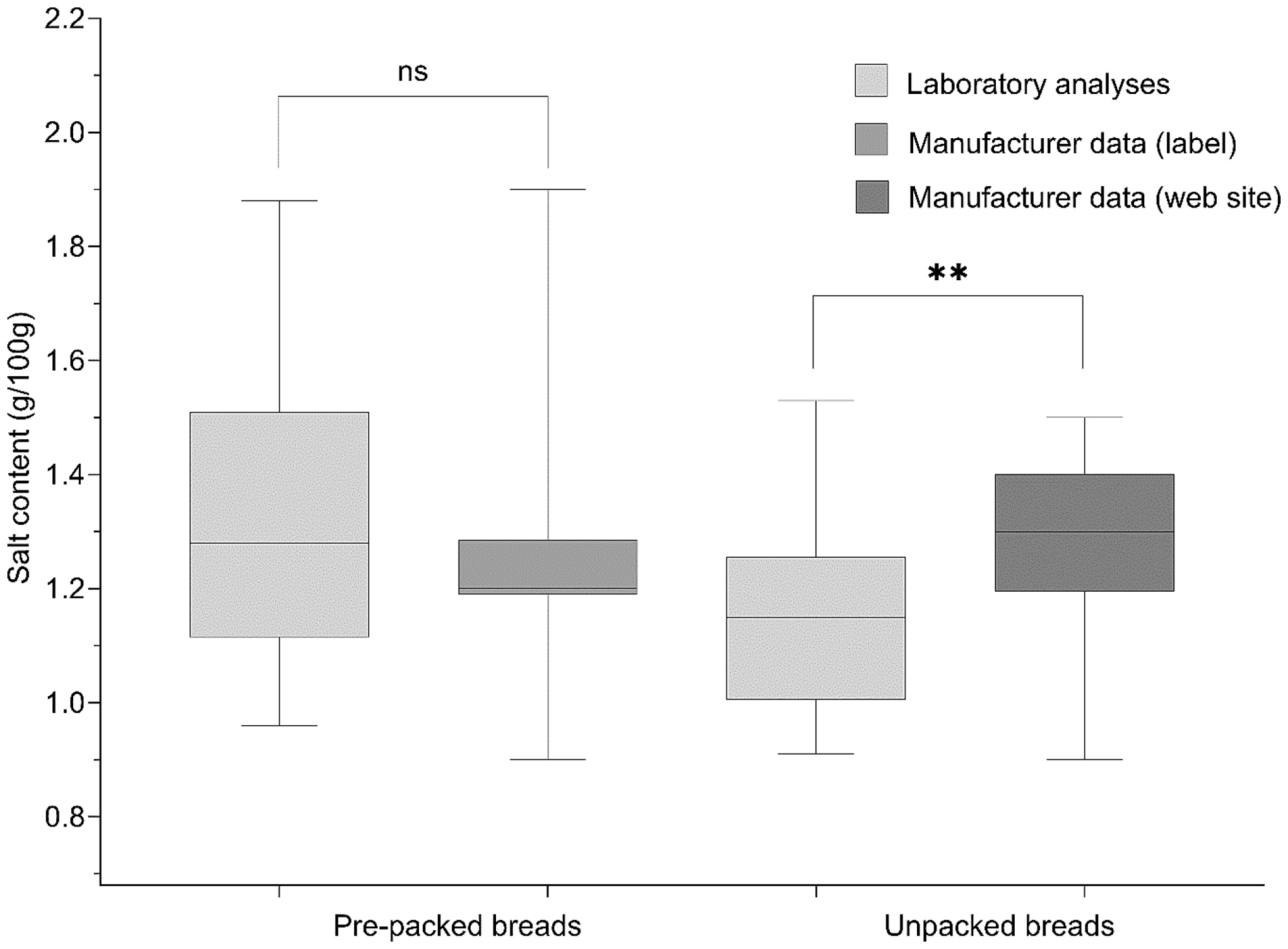
Box-and-whisker plots comparing salt content using laboratory analyses and manufacturer data nutrition declarations on food labels or websites of manufacturers/retailers for unpacked and prepacked breads (**represent significance at *p* < 0.01; ns, not significant difference).

For some bread samples purchased in larger retail shops we were able to access manufacturer data on the salt content, either from nutrition declaration on food labels or manufacturer website. Using pair-comparison t-test, we explored the difference between laboratory analyses and manufacturers’ salt content data (Figure 2). We did not observe significant difference between the labeled and analytically determined salt content in prepacked breads, where regulation require mandatory declaration of the salt content. On the other hand, statistically significant difference was found in unpacked breads, where manufacturers’ data on the salt content was taken from non-regulated websites.

## 4 Discussion

White wheat bread is most commonly consumed bread type in Slovenia, and therefore also most critical for food reformulation, because lowering of its salt content would have highest public-health effects. Interestingly we observed a significant difference between salt content in white wheat bread sold in large retail shops and small bakeries, with the latter having higher mean salt content (1.21 vs. 1.34 g/100 g, respectively). The reasons for such difference could be related to consumer preferences in the neighborhoods of specific small bakeries, differences in the manufacturing codes of practices (i.e., small bakeries are not included to Chamber of Commerce and Industry of Slovenia, which organize voluntary salt reduction program in Slovenia for major bread manufacturers), or technological variations which stem from specific characteristics of the used ingredients. These may also in part explain the high salt content range for white wheat bread sold in smaller bakeries (1.21 g/100 g), which is a consequence of very different amounts of salt used in the manufacturing process. A study on artisanal and industrial bread in Italy reported a higher overall salt content of common white wheat bread (1.5 (0.4) g/100 g) than our findings, however, unlike our results the salt content in industrially produced bread in Italy was higher than that of artisanal bread (35). Furthermore, an analysis of salt content in bread sold in the capital of neighboring Croatia indicated even higher salt levels, with the average content in white wheat bread reaching 2.00 (0.03) g/100 g in larger industrial bakeries and 2.23 (0.07) g/100 g in smaller bakeries (36). Similar results were also observed in Spain and Poland, where the average salt content in white wheat bread was determined at 2.09 (0.3) g/100 g and ~ 2.29 g/100 g, respectively (37, 38). Interestingly, the average salt content in wholegrain wheat bread was slightly higher than in white, half-white or mixed wheat bread. This is in line with results from a study done in Poland (38). Nonetheless, the variability in salt content of this bread type was high and could be a consequence of the use of different ingredients (e.g., using higher amount of salt to improve taste and aroma of wholegrain bread) and technologies, or variations in sodium content of different wheat flour types (39). It should be also noted that our sample of wholegrain breads in large retail shops was rather small, because of very limited offers of wholegrain breads in Slovenia. Analytically measured salt content in our samples of dark wheat bread and rye bread showed a lower salt content than other types (1.15 g/100 g and 1.18 g/100 g, respectively). This is almost half of salt content of dark wheat bread from bakeries in Zagreb (Croatia) where it was determined to be between 2.51 and 2.56 g/100 g (36). For rye bread, our results indicate lower mean content than that of both wholemeal rye bread and mixed wheat-rye bread from Poland (38).

We also observed significant differences between analytically measured salt content in un-packed breads, compared to manufacturers’ salt content data published on websites, where nutrition declaration data can be provided voluntarily also for non-prepacked foods. We found that manufacturers provided data on higher salt content that was actually found in bread samples, which might be explained with the fact that on-line data is not regularly updated, even if foods are being reformulated. On the other side, we did not find such differences on sample of prepacked foods, where manufacturers data on the salt content was taken from mandatory nutrition declaration of food label. While in our study food labels were quite reliable, this cannot be projected to other jurisdictions, with different manufacturing practices and regulations. For example, Bernardo et al. compared sodium content determined by laboratory analysis and in nutrition declaration on bread rolls in Brazil and highlighted notable differences between −5 and 23% for rolls made from proprietary recipes, and between 16.1 and 46.9% in processed dough bread rolls (40). The observed difference between analytically measured values and nutrition declaration values could be due to different methods used to determine them. According to EU legislation, the labeled values should, according to the individual case, be average values and based on either the manufacturer’s analysis of the food; a calculation from the known or actual average values of the ingredients used; or a calculation from generally established and accepted data (41).

Between 2010 and 2020, Slovenia implemented a national action plan to reduce salt intake in the Slovenian population (26). The plan included voluntary targets for various food categories such as bread and bakery products, meat products, salty snacks and breakfast cereals, which were based on the targets set in the United Kingdom. However, previous monitoring of bread in 2012 showed that the targets were not met, and a similar conclusion can be drawn for our results for year 2022. Furthermore, in 2021 the WHO introduced global sodium benchmarks for various food categories. Specifically, a target value of 330 mg sodium/100 g was set for leavened bread made from any type of cereal flour, which corresponds to approximately 0.84 g salt/100 g (42). Unfortunately, none of the analyzed breads in 2022 met this target, which highlights the need for further actions on reformulation and lowering the salt content.

Salt (sodium chloride) is an important ingredient in bread and bakery products as it stabilizes the gluten structure, modulates the fermentation rate, improves the color of the crust and texture and reduces water activity, which prolongs shelf life (43). Gradually reducing the salt content in bread to a set target is considered as the most common strategy. Studies on acceptability of reduced-sodium bread have reported that sodium reductions of up to 30–50% are acceptable to consumers (44–46). However, simply reducing the amount of salt used could have certain disadvantages such as the requirement of other producers’ compliance to lower salt content or the possibility of consumers to simply purchase bread that does not have reduced salt content (47), which also indicates the importance of consumer education for the success of reducing salt content and intake. Furthermore, reducing salt content beyond a certain point and without other additives/substitutes or production modifications, can lead to uncontrolled fermentation, changes in sensory profile, pale color of the crust due to an insufficient Maillard reaction, and a shorter shelf life, as salt also plays an important role in food safety. Since it reduces water activity and thus limits microbial growth, lowering the salt content requires careful adjustments with other strategies (48). In addition, reducing salt content can also lead to production difficulties due to increased dough stickiness. The mitigate such effects, replacing a proportion of sodium chloride with other salts is possible. Among them, potassium chloride seems to be promising, as potassium helps to maintain a similar dough rheology to doughs with sodium chloride (49). According to results of several studies, replacing ~25–50% of sodium chloride with potassium chloride is possible without decreasing product quality or increasing production difficulties. Other strategies for lowering the salt content of bread have also been explored, such as using taste enhancers (e.g., yeast extracts, monosodium glutamate, etc), creating taste contrasts by spatially distributing different salt concentrations (e.g., using two bread doughs with different concentrations), using coarse-grained salt, or modifying flavor by adding other herbs and spices (23, 47).

We used weighting approach to provide insights about changes in the salt content in the general category of bread in Slovenia. With consideration of statistical data on the consumption of various bread types, weighting approach was used to construct salt content for typical bread in Slovenia. This approach indicated a 7% decrease in salt content in bread category between the years 2012 and 2022 (Year 2012: 1.35 g/100 g; year 2022: 1.26 (95% CI 1.22–1.29) g/100 g). Considering very long observation period, we could label such progress as very conservative. This is particularly evident, when such a progress is compared to some other countries. For example, Brinsden et al. reported that, on average the salt content in packed bread in the United Kingdom has fallen by 20%, from 1.23 ± 0.19 g salt/100 g in 2001, to 0.98 ± 0.13 g salt/100 g in 2011 (24). In a recent report on the percentage of food categories meeting the salt reduction targets (average and maximum) for 2017, it was reported that 95% of in-home and 54% of out-of-home bread and roll products were at or below the maximum target of 0.45 g sodium/100 g (or approx. 1.14 g salt/100 g) (50).

While salt content of bread is relatively low, bread represents a staple food and is one of the main sources of salt in most European countries (19). In a nationally representative study of dietary habits and intake in Slovenian population, it was estimated that adult men consume an average of 177 grams of bread per day, while women consume 119 grams per day (51). Considering our results on the mean salt content of typical bread sold in Slovenia, this consumption corresponds to an estimated daily salt intake of about 2.2 grams for men and 1.5 grams for women. Compared to the very recently published data on salt consumption of Slovenian adults based on urinary sodium concentration (52), these values would represent about 19% of the daily salt intake for men and 17% for women. Furthermore, compared to the recommended daily salt intake of less than 5 grams, salt intake from bread accounts for about 30 to 46% of the recommended intake, respectively.

Our study indicates that activities to reduce the salt content in bread in Slovenia should be strengthened in future. This is even more important for smaller bakeries which are not included in voluntary salt reduction program, organized within Chamber of Commerce and Industry of Slovenia and which aims to gradually reduce the salt content of bread and bakery products. This program’s first target was 5% reduction of the salt content by the end of 2022. However, no new targets have been set since then and therefore the results of our study will provide an important basis for future reformulation strategies and reduction targets in Slovenia. Additionally, it would be interesting to examine the salt reduction strategies large producers and smaller bakeries themselves use and their opinions on it, as it could provide better insight and starting point for the next reformulation strategy.

While a reduction in the salt content of bread could potentially have an impact on the overall salt intake of the Slovenian population, it would also be interesting to investigate how this affects iodine intake. In countries such as the Netherlands, it was shown that bread makes an important contribution to adequate iodine intake (53), as the salt used in the production of bread is iodized. In addition, an investigation of the changes in sodium and potassium intake in the case of a (partial) substitution of salt with KCl in bread would be informative, as would an economic evaluation of such a strategy. Furthermore, with consideration of wide sampling approach, our study enables further longitudinal observation of changes of the composition of bread in Slovenian food supply, both in large retailers and small bakeries.

### 4.1 Strengths and limitations

Our study included the main categories of bread available in both large retail shops and smaller bakeries in the 11 statistical regions of Slovenia. The salt content of bread in Slovenia has not been evaluated before to this extent. The sodium (salt) content of bread samples was analyzed by chemical analysis with ICP-MS, which has the potential to yield accurate results for foods with lower sodium concentrations. This approach also provides valuable insight, especially considering that nutritional information for unpacked breads is usually not displayed to the consumers at the point of purchase. Another strength of the study was that all samples were purchased in the real-life retail environment and not provided by the manufacturers. This approach assured us that we analyzed samples of bread, which are indeed available to consumers.

However, we should also mention some study limitations. Although we used a weighing approach to mitigate bias due to sampling, it cannot be completely ruled out as we asked the bakery worker to choose bestselling white and wholegrain bread. The sample size for certain bread categories (e.g., wholegrain bread) was relatively small and with high variability in the salt content; therefore, we could not explore the differences between different sale points in depth. At this point, we should mention that the small sample size is due to the limited availability of wholegrain bread in large retail stores in Slovenia. We should also mention that the bread samples were purchased as they were offered and may deviate from the quality standards for bread set by law in Slovenia (e.g., wholegrain bread with lower percentage of wholegrain flour than defined in the Rules on quality of bakery products (32)). This limitation is particularly relevant for the sample of breads from small bakeries, where some of the wholegrain breads may actually be categorized as mixed breads, but we did not have access to ingredient lists to verify this. We should also mention that different laboratory analytical methods were used for determination of salt content in years 2012 and 2022, and therefore comparisons between our results from 2012 and 2022 should be interpreted with caution. This also applies to comparisons with the results of other countries, as many used the potentiometric or Volhard method to determine the salt content, which can be less accurate as the ICP-MS method used in our study for year 2022. Additionally, we calculated the salt content on the assumption that all sodium corresponds to sodium chloride. However, sodium may also have been added in the form of other sodium salts (e.g., sodium carbonates), which could lead to a slight overestimation of the salt content.

## 5 Conclusion

Study results showed small decrease in the salt content in bread category in Slovenia in the last decade. The decrease was somewhat more notable in the subcategory of white wheat bread sold in large retail shops. However, the targets for sodium/salt reduction set by the WHO have not been met. A large variability between bread samples has also been observed. This study provides an important insight into salt content reduction progress and emphasizes the importance of strengthening activities on the reformulation of bread. This is even more important for smaller bakeries, which are currently not efficiently included into voluntary bread reformulation program.

## Data Availability

The raw data supporting the conclusions of this article will be made available by the authors, without undue reservation.
