# Supplementary material for: Insights into the salt levels in bread offers in Slovenia: trends and differences
Source: Front Nutr. 2025 Jan 14;11:1473362. doi: 10.3389/fnut.2024.1473362 (PMC11772098; doi:10.3389/fnut.2024.1473362)
Supplement: Supplementary file 1 [file Table_1.docx]

Supplementary Material

# Supplementary Tables

**Supplementary Table 1.** Description of the samples and individual consumption of bread and sampling weights according to SURS household consumption data for 2010 and 2018 (27).

| **Type of breads** | | **Total** | White | Half-white | Dark | Mixed | Rye | Wholegrain | Corn | Small bread rolls | White bread toast | Other types of bread |
| --- | --- | --- | --- | --- | --- | --- | --- | --- | --- | --- | --- | --- |
| **Household consumption report bread and types** | |  |  |  |  |  |  |  |  |  |  |  |
| 2010 | kg/individual/year | 29.5 | 10.8 | 4.4 | 3.5 | 2.5 | 1.1 | 0.6 | 0.4 | 3.9 | 1.7 | 0.6 |
|  | share per total (%) | 100 | 36.4 | 14.8 | 11.8 | 8.4 | 3.7 | 2.0 | 1.3 | 13.3 | 5.6 | 2.8 |
| 2018 | kg/individual/year | 24.0 | 6.4 | 2.4 | 2.8 | 2.0 | 0.5 | 0.6 | 0.6 | 3.9 | 1.7 | 8.8 |
|  | share per total (%) | 100 | 26.7 | 9.9 | 11.7 | 8.1 | 2.1 | 2.4 | 2.5 |  |  | 36.7 |
| **Samples** | | |  |  |  |  |  |  |  |  |  |  |
| 2012 | Larger retail stores | | 13 | 9 | 6 | 13 | 3 |  |  |  |  |  |
|  | Small local bakeries | | 1 |  |  | 3 |  |  |  |  |  |  |
|  | **Total** | | **14** | **9** | **6** | **16** | **3** |  |  |  |  |  |
|  | % from total | | 31.1 | 20.0 | 13.3 | 28.9 | 6.7 |  |  |  |  |  |
|  | % within large retail stores | | 34.1 | 22.0 | 14.6 | 31.7 | 7.3 |  |  |  |  |  |
|  | weights | | 1.26 | 0.80 | 0.95 | 0.25 | 0.60 |  |  |  |  | **NA** |
| 2022 | Larger retail stores (count) | | 28 | 12 | 16 | 22 | 3 | 4 | 7 | 15 | 10 |  |
|  | Small local bakeries (count) | | 32 |  |  |  |  | 29 |  |  |  |  |
|  | **Total N** | | **60** | **12** | **16** | **22** | **3** | **33** | **7** | **15** | **10** |  |
|  | % from all samples | | 33.7 | 6.7 | 9.0 | 12.4 | 1.7 | 18.5 | 3.9 | 8.4 | 5.6 |  |
|  | % from large retail stores | | 23.9 | 10.3 | 13.7 | 18.8 | 2.6 | 3.4 | 6.0 | 12.8 | 8.5 |  |
|  | weights | | 0.63 | 1.17 | 1.03 | 0.52 | 0.98 | 0.10 | 0.51 | 1.53 | 0.97 | **NA** |

Notes: NA, not applicable.

**Supplementary Table 2**. Unweighted and weighted data for salt content in breads purchased across Slovenia in 2012 and 2022.

|  | 2012 | | 2022 | |
| --- | --- | --- | --- | --- |
|  | N (%) | Mean (95% CI) salt content (g/100g) | N (%) | Mean (95% CI) salt content (g/100g) |
| Unweighted data | 45 (100) | 1.35 (1.30-1.41) | 178 (100) | 1.27 (1.24-1.31) |
| Weighted data |  | 1.35 (1.28-1.42) |  | 1.26 (1.22-1.29) |

CI, Confidence interval. Weighting of the data was conducted to avoid bias due to the sampling technique; it considered the most recently available data for certain sampling year from the Statistical Office of Slovenia on consumption of breads in particular bread category in Slovenian households.
